# Spiritual Beliefs, Mental Health and the 2019 Coronavirus (2019-nCoV) Outbreak: What Does Literature Have to Tell Us?

**DOI:** 10.3389/fpsyt.2020.570439

**Published:** 2020-10-30

**Authors:** Jucier Gonçalves Júnior, Jair Paulino de Sales, Marcial Moreno Moreira, Carlos Kennedy Tavares de Lima, Modesto Leite Rolim Neto

**Affiliations:** ^1^Santa Casa de Misericórdia de Fortaleza, Fortaleza, Brazil; ^2^University Catholic Center of Quixadá, Ceará, Brazil; ^3^Faculty of Medicine, Federal University of Cariri, Ceará, Brazil; ^4^Faculdade de Medicina do ABC, São Paulo, Brazil

**Keywords:** coronavirus infection, mental health, spirituality, coping, public health

## Introduction

On December 2019, when the β-coronavirus 2019-nCoV (COVID-19) pandemic started in Wuhan, China (1, 2) the social relations were completely changed. Due to the fact that several key characteristics of the transmissibility and natural history of COVID-19 are currently unknown (3) and the dubious or even false information about factors related to virus transmission, the incubation period, its geographic reach, the number of infected, and the actual mortality rate has led to insecurity and fear in the population (4).

The literature suggests that the Spirituality is a common way that people cope with illnesses (5) and there are papers reporting that 65 percent of patients with depression, anxiety, and other psychiatric conditions indicate that they want spirituality to play a part in their treatment (6). However, the use of Spirituality to deal with the COVID-19 pandemic is still uncertain. Therefore, the objective was to describe the impact of these strategies to promote mental health in psychiatric patients.

## Discussion

Epidemiological data from developing countries suggest that Spirituality evokes in health-care receivers the sources to find the necessary inner strengths, which include perspective thinking, rituals for transcending immediate physical condition, and modalities of coping with their illnesses (7). In COVID-19 context, spirituality can be a tool to face the negative symptoms that appear in general community and psychiatry patients. However, the influence of spirituality on mental health is a phenomenon resulting from several factors such as: lifestyle, social support, a belief system, religious practices, ways of expressing stress, spiritual direction, and guidance (8). Thus, some groups could benefit more from spiritual practices than others, such as those who use drugs, those with mood or anxiety disorders and compensated psychotic disorders as long as they obviously have belief in the rituals practiced.

Spirituality, during the COVID-19 pandemic, can assist in the prevention and adjuvant treatment of psychiatric illnesses by giving meaning and resignifying the patient's life; positively reinforce their beliefs in the quest to improve themselves; assist in overcoming one's own limits, increasing adherence to treatment and implementing strength at will (9). This positive reinforcement is essential, as it allows the patient to maintain a center of balance, optimism and hope and, therefore, to continue with the proposed treatment without “relapses” or unfavorable developments. Thus, another of the possible benefits would be an improvement in the self-care of psychiatric patients, which would denote less demand for health services and, therefore, less exposure. It is important to remember that people with mental disorders can be exposed to more barriers in accessing timely health services, because of discrimination associated with mental ill-health in health-care settings (10).

On the other hand, people with mental health conditions could be more substantially influenced by the emotional responses brought on by the COVID-19 pandemic, resulting in relapses or worsening of an already existing mental health condition because of high susceptibility to stress compared with the general population (10, 11). Strategies associated with thought based on faith and hope can provide a search for the reframing and for the momentary relief of the suffering, based on spiritual values and religious beliefs (12). Among the strategies to deal with negative symptoms raised during the pandemic, we can mention: reflections and readings of religious texts; online prayer meetings and exchange of experiences; online support groups made possible by churches / temples; and meditation. Thus, spiritual practices can guide psychiatric patients in the search for self-knowledge, self-control and assist in the creation of autonomous practices (8) in the management of their pathologies and the balance between underlying diseases and situations inherent to the pandemic itself.

Another determining factor is the capacity that spirituality has to assist in the elaboration of the concept and coping with grief by the individual, extremely present during the COVID-19 pandemic. Literature review showed that patients with spiritual practices find it easier to deal with losses and face the mourning of loved ones in a more harmonious way than those who do not, improving their quality of life (13). Besides that, spirituality evokes hope in patients and optimizes the quality of life by withstanding negative symptoms. As a result of a study conducted in Nepal, hope was considered a predictive factor of quality of life, since the increase in the levels of hope culminated in improving the quality of life of the patients studied (14).

Although quarantine is an effective mean, thus far, in the management of COVID-19 pandemic it can predispose to isolation, feeling of abandonment and deepens whether depressive, anxiety (including panic attacks and post-traumatic stress), psychotic or paranoid, and can even lead to suicide (4). In this context, Spirituality appears to be beneficial in improving coping abilities in people with mental disorders as well as in reducing suicidality. A study carried out in the USA with 472 patients with post-traumatic stress disorder showed that religious/spiritual practices, regardless of sex, age group or ethnicity are protective factors in the fight against suicidal ideation (15). Similar findings were found in an Indian paper carried out with 160 university students (16). Research carried out in Switzerland with schizophrenic patients, 3 years after the diagnosis, showed that religion/spirituality was understood as a way of coping with illness (17). In the United Kingdom, when analyzing 7,403 participants in an attempt to relate the prevalence of mental disorders and religious practices, among those who had no spiritual practice there was greater use of psychotropic medications, 95% complained of phobia or anxiety, 73% had already had some experience with drugs in life, and 56% reported not eating properly (18).

Spirituality is a source of strength and comfort, helps with relieving psychiatric symptoms and it is associated with reduced length of hospital stay and improved satisfaction with life (19). A research carried out with 881 hospitalized patients over 50 years of age demonstrated that the organization of religious activities had a positive impact, decreasing the length of hospital stay (20). Meta-analyzes demonstrated that 30-90% of people with schizophrenia or severe mental illnesses considered spirituality to be an essential resource for dealing with difficult/stressful events (21).

Spirituality is also a strength tool for caregivers to deal with their emotions and improve care for the psychiatry patients. According to Weaver, Flannely and Oppenheimer, family caregivers also rely heavily upon their religious faith to cope with the burden of caring for their loved ones (5). Brazilian studies with caregivers and patients with chronic diseases show that Spirituality, as a strategy for coping, becomes an important ally when dealing with difficulties, being an important form of support and well-being for caregivers (12).

Therefore, during the COVID-19 pandemic, which already counts 976,249 cases and 50,489 deaths in 207 countries (22), spirituality has the potential to reinforce the patient's positive symptoms (5, 12, 13, 21) in relation to the disease, to the way they deal with limitations or problems during the pandemic, optimize their therapeutic adherence (8, 9) to pharmacological and non-pharmacological approaches, but also empowering the patient in other spheres of their life, such as emotional (improving self-esteem and self-control); social—expanding their friendship circle and strengthening family ties; and intellectual, as it engages them in activities such as reading, singing, meditation, and oratory (13, 17, 19), reducing the negative effects of the pandemic, social isolation and routine deprivation, deeply necessary to establish a sense of security and normality. However, these benefits only seem to be demonstrated in patients with well-established spiritual ties/practices and willing to believe/perform the recommended rituals. Additionally, patients with psychiatric disorders such as severe psychoses or in intensive care units in sedoanalgesia, whose connection with reality has been interrupted in some way, would not be able to enjoy the aforementioned benefits, being relegated this opportunity to their families and caregivers. Thus, evaluating spirituality as a tool that provides comprehensive and more assertive care is essential for effective psychiatric and psychological care and is particularly urgent in COVID-19 times.

## Author Contributions

MLRN, JGJ, CKTL, and MMM reviewed the study protocol. JPS read and screened articles for inclusion. All authors critically reviewed drafts and approved the final manuscript.

## Conflict of Interest

The authors declare that the research was conducted in the absence of any commercial or financial relationships that could be construed as a potential conflict of interest.
